# Dielectric Permittivity Measurement Using Open-Ended Coaxial Probe—Modeling and Simulation Based on the Simple Capacitive-Load Model

**DOI:** 10.3390/s22166024

**Published:** 2022-08-12

**Authors:** Antonio Šarolić, Anđela Matković

**Affiliations:** FESB, University of Split, HR-21000 Split, Croatia

**Keywords:** dielectric permittivity measurement, open-ended coaxial probe, electromagnetic modeling and simulation, capacitive-load model, sodium chloride (NaCl) water solution, saline ionic conductivity, physiological saline, CST, FEKO, reflection coefficient de-embedding, microwave frequency range

## Abstract

The study aim was to validate that dielectric permittivity measurement using the open-ended coaxial probe can be reliably modeled using electromagnetic modeling and simulations, followed by the postprocessing calculations based on the simple capacitive-load model. Saline solutions with various NaCl concentrations were used as materials under test (MUTs) to investigate how ionic conductivity affects the model validity. Two different solvers and simulation methods were used: FEKO for the frequency domain and CST for the time domain. Furthermore, we performed physical experiments with the same probe and MUTs, again implementing the capacitive-load model on the measurement data to observe the model validity. Relative error of the capacitive-load model with respect to the reference permittivity values, both in measurements and simulations, was within 10% for all cases except for the measured $\varepsilon_{r}^{\prime }$ of 1M solution at the lowest frequencies. The model yielded average relative errors well below 1% for the physiological saline, which is relevant for biological materials. The error increased for higher concentrations and for the lowest simulated frequencies but was within the declared measurement accuracy of the probe itself. This makes the simple capacitive-load model valid for all analyzed concentrations in the microwave frequency range from 0.5 to 18 GHz.

## 1. Introduction

Dielectric permittivity of a biological tissue is the key parameter determining its interaction with the electromagnetic field in the radiofrequency (RF) and microwave (MW) frequency range [1]. Knowledge on dielectric permittivity is hence required to analyze how biological tissue responds to the imposed RF or MW electromagnetic field, either during biomedical procedures involving electromagnetic field applications or during inadvertent exposure. Systematic sets of results have been reported in [2,3,4,5]; however, these sets provide only limited data and are continuously challenged by newly reported results. In bioelectromagnetics, there is a constant need to perform more accurate measurements, to increase frequency range and resolution, to measure tissues at different temperatures, and to measure malignant and diseased tissues. In sensor applications, dielectric permittivity is measured in biological and non-biological materials to indirectly determine the concentration of a chemical ingredient that significantly affects the permittivity, such as water, salt, or glucose (e.g., [6,7,8,9,10]). In wireless communications, dielectric permittivity is a crucial parameter for analyzing electromagnetic wave propagation in materials (e.g., [11]). All of this makes dielectric permittivity measurement an important procedure that is used in various industries and that will be used even more as the associated measurement methods overcome their inherent limitations.

Lots of applications require the measurements to be non-destructive, which limits the possible designs of dielectric probes. When there is an additional requirement that the measurement must be broadband, the open-ended coaxial probe is the favored and widely accepted design [12,13,14]. It is based on the coaxial line, an inherently wideband structure, and the sensing is achieved by measuring the reflection from the probe open end which is only in surface contact with the material under test, making this method non-destructive.

In practical use, the coaxial probe dielectric measurement suffers from several uncertainties, a few of which are mentioned here. The probe response, although inherently wideband, is actually frequency-limited from both sides, meaning that the measurement error increases as the frequency decreases below or increases above the working frequency range [15]. The probe response to inhomogeneous materials is very complex and often unknown [16,17,18,19]. When measuring layered materials, the result depends on the probe sensing depth, which should be determined for the used probe [20,21,22,23]. When measuring biological tissues, the result also depends on the contact pressure, which should be regarded during the measurements [24,25]. The accuracy of the final measurement result additionally depends on the postprocessing method, which in turn depends on the chosen model of the open-end load impedance [15,26,27,28,29].

Consequently, these uncertainties and potential errors translate to the limitations of the coaxial probe, which can only be overcome by thorough analyses of various constraint factors. Such studies could be performed by physical experimentation; however, this is often demanding when experimenting in a wide space of parameters. Therefore, in order to facilitate such studies, it would be very useful to reliably model and simulate the complete measurement process, thus moving from physical experiments to computer simulations. Since the probe is measuring electromagnetic phenomena, the response of the probe is first determined by the electromagnetic modeling and simulation. Dielectric permittivity is then obtained from this response by postprocessing the results of electromagnetic simulation. Postprocessing is again based on modeling the actual load impedance even in an actual physical measurement process. The accuracy of this entire modeling and simulation therefore depends on the accuracy of the initial electromagnetic model, selected numerical method for electromagnetic simulation, and the selected postprocessing calculation model. Accordingly, modeling and simulation of the complete measurement process should be carefully prepared, performed, and validated in order to be used for relevant coaxial probe analyses.

The main aim of this paper was to demonstrate and to validate that the dielectric permittivity measurement method using open-ended coaxial probe in the microwave frequency range can be completely and reliably modeled and simulated using computer electromagnetic modeling and simulations followed by the postprocessing calculations based on the simple capacitive-load model within the typical measurement accuracy of the probe itself.

The previous state-of-the-art [15,26,27,28,29,30] suggests that the simple capacitive load successfully models the actual load if the frequencies are not as high as to cause radiation from the probe into the MUT. Accordingly, the validity of this model should be examined with respect to the probe dimensions, frequency range, and MUT characteristics.

The open-ended coaxial probe has been extensively electromagnetically modeled and simulated before; however, each study had its own choices of open-ended coaxial probe models, simulation methods, MUTs, and frequency ranges (e.g., [20,21,30,31,32]). In this study, we modeled the flangeless slim-form probe manufactured by Keysight Technologies. This choice was based on its convenience and the common acceptance for measurements of biological tissues due to its slim form and the absence of flange. This exact probe design is unique, considering the commercial probes in this frequency range. The probe technical characteristics are presented in Section 2.4. The frequency range of up to 18 GHz analyzed in this study is a considerable extension with respect to the previous studies, enabling the analysis of phenomena occurring at higher frequencies. The postprocessing calculations were based on the simple capacitive-load model, and the aim of this study was to analyze the error occurring when using such a model for postprocessing of simulation data in dependence on the ionic concentration of the MUT in the frequency range up to 18 GHz. This was done using sodium chloride (NaCl) solutions in deionized water with various NaCl concentrations as MUTs.

Moreover, two simulation methods and solvers were used to enable comparison between them, which was also one of the aims of this study. Furthermore, we also performed physical experiments with the analyzed exact slim-form probe and MUTs, again implementing the simple capacitive-load model on the measurement data, to observe the overall validity of such a postprocessing model when used to postprocess either measurement or simulation results.

The paper structure follows the study organization. Materials and methods are presented in Section 2, with subsections covering: the concept of the complete investigation; dielectric permittivity measurement method using open-ended coaxial probes in general; flangeless slim-form probe as the object of this study; description of materials under test; description of measurement parameters, description of simulation parameters, and description of the simple capacitive-load model. The results are presented in Section 3, with subsections for the measurement and the simulation results. The overall findings are further discussed in Section 4, with the conclusions given in Section 5.

## 2. Materials and Methods

### 2.1. Study Concept

#### 2.1.1. Validation of the Simple Capacitive-Load Model by Physical Measurements

The first part of the study, i.e., validation of the simple capacitive-load model by physical measurements, examined how accurate the simple capacitive-load model is for modeling the coaxial open end terminated by a material under test (MUT), i.e., how the measurement results obtained by the simple capacitive-load model agree with the measurement results obtained by a more complex load model embedded in the manufacturer measurement software [33]. Dielectric permittivity of water and several saline solutions were physically measured by the open-ended coaxial probe. Generally, the measured permittivity value is obtained by postprocessing the raw measurement result, i.e., the measured open-end reflection coefficient. In this study, two methods were used for postprocessing, and the results were compared and discussed. The first method, regarded hereby as accurate and thus serving as a reference for comparisons, is the one embedded in the manufacturer-provided measurement software and uses a proprietary load model to calculate the complex permittivity from the reflection coefficient. The complex permittivity was then also calculated from the reflection coefficient by our own postprocessing calculations using the simple capacitive-load model, as the second method. The permittivity values obtained by the two methods were compared to check both the validity and the limitations of the hypothesis that the load could be modeled using a simple capacitive-load model. Finally, the measured permittivity values were additionally compared to the reference values of Debye and Cole–Cole dispersions (water and saline solutions, respectively), in order to verify the probe behavior and the overall validity of the measurement setup.

#### 2.1.2. Validation of the Simulation of the Complete Measurement Process

The second part of the study, i.e., validation of the simulation of the complete measurement process, examined the agreement of the coaxial probe simulation results obtained by two entirely different numerical methods applied on the same probe model, followed by identical postprocessing calculations. Not only do the two numerical methods belong to two different software packages but they are also different in their approaches to the electromagnetic problem: one solves it in the frequency domain and the other one in the time domain. Method of moments (MoM) was chosen as a frequency-domain solver implemented in FEKO [34] and Finite Integration Technique (FIT) as a time-domain solver implemented in CST [35]. The slim-form probe was modeled first, then the MUT volume was added to the probe model. MUT permittivity was set to the value for water and then to the values for several saline solutions, in accordance with Debye dispersion for the water and Cole–Cole dispersion for the saline solutions. Then, the electromagnetic simulations were performed for each MUT, obtaining the reflection coefficient at the probe input port. Finally, we calculated the MUT permittivity from the reflection coefficient using the simple capacitive-load model. The calculated MUT permittivity, as the final output of the overall simulation process, was compared to the MUT permittivity initially set in the software model (Debye and Cole–Cole dispersions) in order to validate the modeling and simulation method as a whole.

The results were compared and discussed, allowing us to conclude the validity of the proposed method for modeling and simulation of the open-ended coaxial dielectric probe measurement method in the microwave frequency range based on the simple capacitive-load model.

### 2.2. Dielectric Permittivity

The dielectric permittivity $\hat{\varepsilon }$ of the material (in farads per meter, F/m) is a physical quantity that describes the response of the material to the imposed electric field. It is a complex quantity consisting of the real part $\varepsilon^{\prime }$, describing the polarization response of the material, and the imaginary part $\varepsilon^{\prime \prime }$, describing the losses in the material as the response to the imposed electric field. A material can be described by its relative permittivity $\hat{\varepsilon }$_r_ (hereafter written simply as $\varepsilon_{r}$), a dimensionless complex quantity which when multiplied by the vacuum permittivity $\varepsilon_{0}$ (in F/m) yields the complex permittivity $\hat{\varepsilon }\;$(hereafter written simply as ε):

(1) $$\hat{\varepsilon }=\varepsilon^{\prime }-j\varepsilon^{\prime \prime }=\varepsilon_{0}\cdot \left(\varepsilon_{r}^{\prime }-j\varepsilon_{r}^{\prime \prime }\right)=\varepsilon_{0}{\hat{\varepsilon }}_{r}\;\;.\;$$

Therefore, the actual quantities being measured are the real and the imaginary part of the relative permittivity: $\varepsilon_{r}^{\prime }$ and $\varepsilon_{r}^{\prime \prime }$, respectively. Their ratio is often referred to as the loss tangent tan*δ*, denoting the measure of losses in the material at a specific frequency:

(2) $$\tan\delta =\frac{\varepsilon \prime \prime }{\varepsilon \prime }=\frac{\varepsilon_{r}^{\prime \prime }}{\varepsilon_{r}^{\prime }}.\;$$

The losses arise from the two different mechanisms of dissipation: dielectric losses arising from continuous reorientation of dipoles in the time-changing field and conduction losses due to electric conductivity of the material arising from free charges, either electrons or ions. Considering that both mechanisms form ohmic losses, they can be unified within a single resistance or conductance in the equivalent load model. Consequently, $\varepsilon_{r}^{\prime \prime }$ is sometimes expressed in terms of the equivalent conductivity σ (in siemens per meter, S/m), which then incorporates both losses:

(3) $$\sigma =\omega \varepsilon_{0}\varepsilon_{r}^{\prime \prime }\;\;\;,$$

where *ω* is the angular frequency.

### 2.3. Open-Ended Coaxial Dielectric Probe Measurement Method in General

The open-ended coaxial probe is usually built as a precisely machined segment of a straight rigid coaxial transmission line. One end features the input port in the form of a coaxial connector, while the other end (the probe tip) is truncated and precisely machined as an open end. The open end is inserted into a MUT whose dielectric permittivity is being measured. The electric field lines form between the electrodes of the open end, penetrating the MUT. Thus, the MUT dominantly contains the electric field and determines the termination of the coaxial line. The resulting reflection coefficient can be measured at the probe input port by the vector network analyzer (VNA). VNA performs a one-port reflection measurement by sending the test signal into the probe input port, measuring the reflection from the probe tip inserted in the MUT in the form of the reflection coefficient *Γ*. The reflection coefficient *Γ* is a complex quantity defined by its real and imaginary part, i.e., by its amplitude and phase.

Considering that *Γ* is a function of $\varepsilon_{r}$ and vice versa, the complex relative permittivity $\varepsilon_{r}$ of the MUT can be derived from the reflection coefficient *Γ* using the postprocessing calculations. These calculations are incorporated in the software provided by the probe manufacturer, accompanying the probe, thus completing the whole measurement method provided to the probe user. The calculation method is based on the solution for the equivalent model of the probe termination load admittance. The probe manufacturers aim for the highest possible accuracy, leading to usage of complex models. Such complex models should preferably include all electromagnetic phenomena occurring in the probe and MUT—if not by analytical modeling of the phenomena, then at least implicitly by finding the best fitting function within the process of probe calibration. Thus, in order to determine the parameters of the load model, the measurement must first be calibrated using three known loads: open (without any MUT, open end is in the air), short (open end is shorted by a conductive sheet), and a known liquid (deionized water being most commonly used, as its dielectric dispersion is well-known).

In order to increase the measurement accuracy, the model should be as rigorous as possible while still allowing the calculations to be performed within a sensible time. The computation speed is an important aspect due to the fact that measurements are commonly performed in a wide frequency span, i.e., in a large number of frequency points, putting a strain on the computation time. Given all the considerations mentioned here, a number of models exist, while the probe manufacturers (such as [36,37]) use the complex ones they consider the most accurate and suitable to their probes. However, the simplest model would be the one taking into account only the capacitive susceptance at the probe open end. One of the aims of this paper was to analyze the validity of this simple model in the microwave frequency range from 0.5 to 18 GHz.

### 2.4. Slim-Form Probe

The object of our study was the slim-form probe—a widely used open-ended coaxial probe manufactured by Keysight Technologies—with all the specifications presented hereafter taken from [36]. It is built as a 200 mm long segment of a straight rigid coaxial transmission line. One end features the input port in the form of a 2.4 mm coaxial 50 Ω connector, while the other end is a flangeless open end having an outer diameter of 2.2 mm, according to the manufacturer specifications.

The inner diameters are not specified by the manufacturer; however, the probe has been commonly referenced [21,38] to conform to the geometry of the RG405 semirigid coaxial cable [39], having a characteristic impedance of 50 Ω. This conformance has been visually confirmed in this study as accurately as possible by the caliper measurements. Small deviations are possible, depending also on the relative permittivity of the cable dielectric, but these are considered too small to affect the modeling accuracy (having an order of magnitude 1/100 of the millimeter). Thus, the probe modeling in this study relied on RG405 dimensions while keeping the characteristic impedance at 50 Ω, as explained later.

The nominal frequency range of the probe from 500 MHz to 50 GHz is specified by the manufacturer [36]. Considering that the upper frequency limit of our VNA is 18 GHz, this study was performed from 500 MHz to 18 GHz. This range still covers most of the microwave applications nowadays, both in biomedicine and in communications.

The maximum frequency *f*_max_ of the probe is further limited by MUT properties according to the following equation given in the specifications [36]:

(4) $$f_{\max\;}<\frac{\left|285-125j\right|}{\sqrt{\left|\varepsilon_{r}\right|}}\;\mathrm{GHz}.$$

A calculation check with the maximum $\left|\varepsilon_{r}\right|$ of MUTs used in this study confirmed that our measurements were not affected by this additional frequency limit.

According to [36], the measurement method with the slim-form probe assumes that the MUT is liquid or soft semi-solid, infinite in size, non-magnetic, isotropic, and homogeneous. It requires a sample size which allows minimum 5 mm insertion and 5 mm around the probe tip. The measured MUT should have $\varepsilon_{r}^{\prime }$ less than 100. These requirements were all satisfied for the MUTs used in this study.

The typical accuracy for both $\varepsilon_{r}^{\prime }$ and $\varepsilon_{r}^{\prime \prime }\;$ is declared in [36] simply as ±10% of the absolute value of the complex permittivity. The accuracy of our measurement results was actually better, as the results deviated less than 10% from the expected Debye and Cole–Cole values of MUTs.

In the probe specifications, the manufacturer does not explicitly disclose the model used for postprocessing calculations to derive the complex permittivity from the measured reflection coefficient. However, the manufacturer application note [40] referenced the paper [41] when mentioning the derivation of the MUT permittivity from the reflection coefficient measured by the coaxial probe, suggesting the use of polynomial model of the load admittance, with optimized coefficients.

### 2.5. Materials under Test

In order to validate the models analyzed in this study, we used well-defined materials whose dielectric properties can be modeled using Debye and Cole–Cole dispersion models: deionized water and sodium chloride (NaCl) solution in deionized water (saline).

Water is commonly used as a reference material, as its dielectric properties have been extensively studied. The frequency dispersion of the complex relative permittivity of water is described using Debye’s empirical equation [42]:

(5) $$\varepsilon_{r}\left(\omega \right)=\varepsilon_{\infty }+\frac{\varepsilon_{s}-\varepsilon_{\infty }}{1+j\omega \tau },$$

where $\varepsilon_{\infty }$ is the relative permittivity at the high-frequency limit, $\varepsilon_{s}$ is the low-frequency or static relative permittivity, and *τ* is the relaxation time of the water. Accordingly, the water permittivity can thus be determined, provided that said parameters are known.

Saline solutions, i.e., sodium chloride (NaCl) solutions in water, can be described by the Cole–Cole empirical equation [43], extended with the ionic conductivity term [44]:

(6) $$\varepsilon_{r}\left(\omega \right)=\varepsilon_{\infty }+\frac{\varepsilon_{s}-\varepsilon_{\infty }}{1+{\left(j\omega \tau \right)}^{1-\alpha }}+\frac{\sigma_{i}}{j\omega \varepsilon_{0}},$$

where *α* is the distribution parameter and $\sigma_{i}$ is the ionic electric conductivity.

Considering that water and NaCl solutions were used as the materials under test in this study, their permittivity could be calculated in advance by Equations (5) and (6), respectively. The calculated permittivity could thus serve as a reference value for simulations. The required Debye and Cole–Cole parameters $\varepsilon_{\infty }$, $\varepsilon_{s}$, *τ*, *α*, and $\sigma_{i}$ for the tested materials could be determined according to the saline concentrations and temperature.

Four saline solutions were used in the study, having NaCl concentrations of 0.154M, 0.25M, 0.5M, and 1M. The concentration of 0.154M refers to physiological saline (0.9% NaCl), commonly referred to as being isotonic to the biological tissue fluids [45], thus well-representing biological materials with high water content. This makes the study and its conclusions relevant and applicable for dielectric studies of biological samples. The other three saline solutions had larger NaCl concentrations, which increased their ionic conductivity, to observe its potential effect on the measurement results.

Dielectric permittivity of water can be described by Debye dispersion (5). We used the following Debye parameters from the well-accepted reference [46] (for 25 °C): $\varepsilon_{\infty }$ = 5.2, $\varepsilon_{s\_\mathrm{water}}$ = 78.36, and $\tau_{\mathrm{water}}$ = 8.27·10^−12^ s to calculate the complex relative permittivity $\varepsilon_{r}\left(\omega \right)$ using Equation (5). Then, we extracted its real and imaginary parts $\varepsilon_{r}^{\prime }\left(\omega \right)$ and $\varepsilon_{r}^{\prime \prime }\left(\omega \right)$ (respectively) to be used as reference values for comparison to the measurement and simulation results.

Dielectric permittivity of NaCl solutions can be described by Cole–Cole dispersion (6), provided that the dispersion parameters $\varepsilon_{\infty }$, $\varepsilon_{s}$, *τ*, *α*, and $\sigma_{i}$ are known. These parameters were calculated according to polynomial equations derived and validated in [44], with the exception of $\varepsilon_{\infty }$ which does not differ between water and the analyzed saline solutions; thus, the value of $\varepsilon_{\infty }$ = 5.2 for water could also be used for all the saline solutions. The model for concentrations below 1M from [44] was used for all four concentrations (0.154M, 0.25M, 0.5M, and 1M), as presented in the following equations:

(7) $$\varepsilon_{s}=\varepsilon_{s\_\mathrm{water}}\left(1-3.742\cdot 10^{-4}tc+0.034c^{2}-0.178c+1.515\cdot 10^{-4}t-4.929\cdot 10^{-6}t^{2}\right)\;\;\;,$$

(8) $$\tau =\tau_{\mathrm{water}}\left(1.012-5.282\cdot 10^{-3}tc+0.032c^{2}-0.01c-1.724\cdot 10^{-3}t+3.766\cdot 10^{-5}t^{2}\right)\;\;\;,$$

(9) $$\alpha =-6.348\cdot 10^{-4}tc-5.1\cdot 10^{-2}c^{2}+9\cdot 10^{-2}c\;\;\;,$$

(10) $$\sigma_{i}=0.174tc-1.582c^{2}+5.923c\;\;\;,$$

where *t* is the temperature in degrees Celsius (25 °C in this case), *c* is the saline concentration in moles per liter (M), and $\varepsilon_{s\_\mathrm{water}}$ and $\tau_{\mathrm{water}}$ are the aforementioned values of static relative permittivity of water and the relaxation time of water, respectively. The resulting values are given in Table 1.

These parameters were input in Equation (6) to calculate the complex relative permittivity $\varepsilon_{r}\left(\omega \right)$ of each saline. Then, we extracted its real and imaginary parts $\varepsilon_{r}^{\prime }\left(\omega \right)$ and $\varepsilon_{r}^{\prime \prime }\left(\omega \right)$ (respectively) to be used as reference values for the simulation models.

The same four saline solutions were used in measurements, with precisely prepared concentrations. The temperature of all samples was kept at the laboratory temperature of 25 °C, additionally stabilized by a water bath.

### 2.6. Measurements

The measurement setup was assembled according to the principles given above in Section 2.3 and Section 2.4. The slim-form probe by Keysight Technologies Inc. (Santa Rosa, CA, USA) [36] was connected to the vector network analyzer FieldFox N9927A (Keysight Technologies Inc., Santa Rosa, CA, USA) by a phase-stable coaxial cable Sucoflex 404 (HUBER+SUHNER AG, Herisau, Switzerland). The VNA was connected to a computer, and the measurements were controlled and performed using the manufacturer-provided software Keysight Materials Measurement Suite N1500A (Keysight Technologies Inc., Santa Rosa, CA, USA) [33]. The room and the MUT temperature were kept at 25 °C, and the MUT temperature was controlled using a precise thermometer DTM3000 (LKM Electronics GmbH, Geratal, Germany).

**Figure 1:**
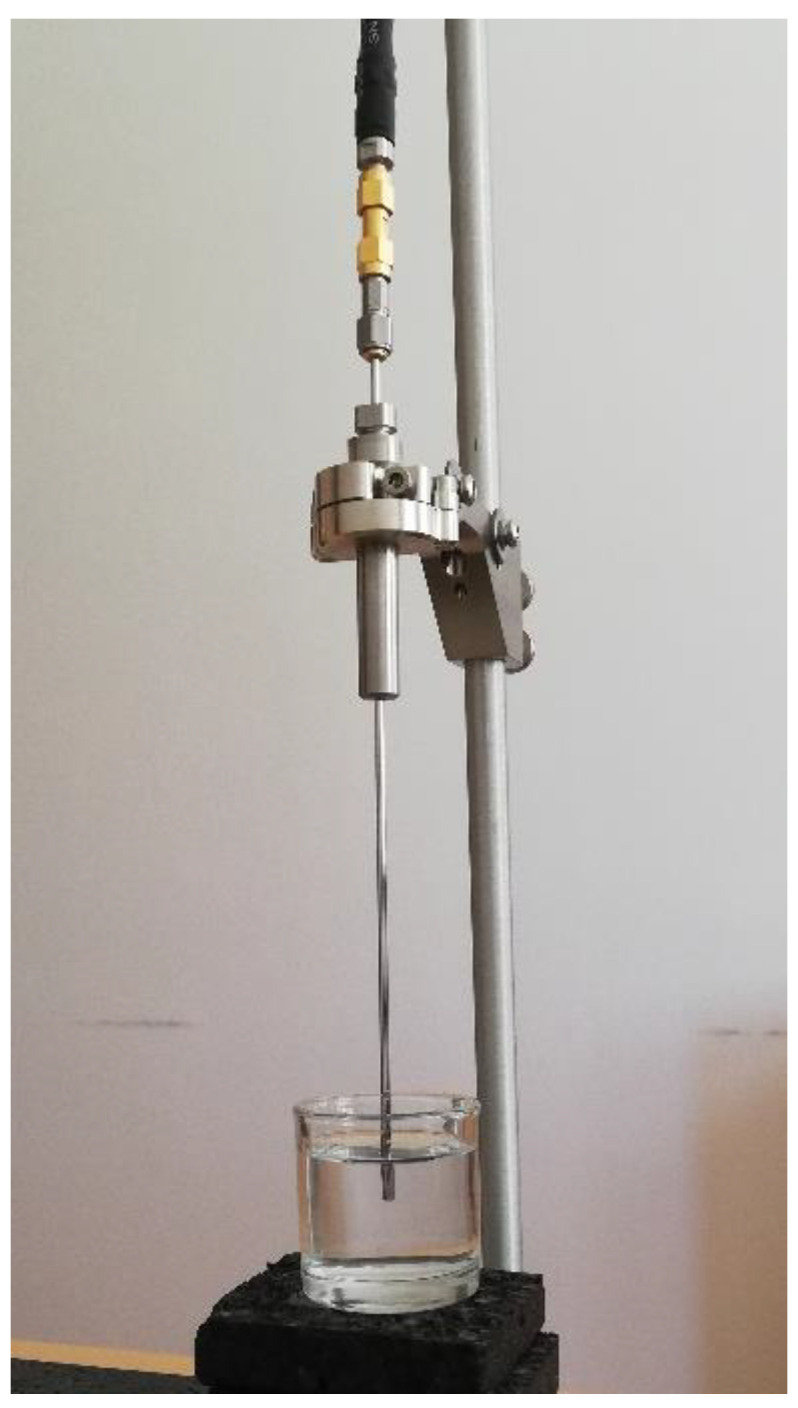
The slim form inserted into a liquid MUT.

The probe was fixed to its stand in the vertical position and inserted into liquid MUT, as shown in Figure 1. The liquid MUT was placed in a glass container, thus assuming the form of a cylinder, 29 mm high and 37 mm in diameter, while the probe insertion depth was kept at 10 mm. These dimensions exceed the aforementioned geometrical requirements given by the manufacturer related to the minimum insertion depth and MUT minimum size. The glass walls of the container did not affect the measurements, which was confirmed both by several pilot measurements with the larger containers, all yielding the same result, and as is later shown, by the results fitting the expected permittivity of the liquids in accordance with their dispersions (Debye dispersion for water and Cole–Cole dispersion for NaCl solutions).

The output power was set to −10 dBm, and the intermediate frequency bandwidth was set to 100 Hz. The frequency range was set to cover the range from 500 MHz to 18 GHz, with linear stepping of 5 MHz. The measurements were calibrated by the open-short-water calibration sequence preceding each MUT measurement.

### 2.7. Simulations

#### 2.7.1. Simulation Model

**Figure 2:**
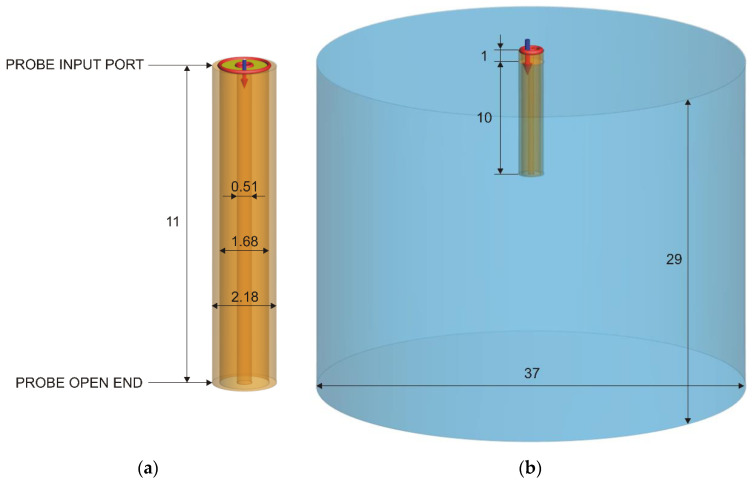
(**a**) The slim-form probe modeled as a coaxial line. (**b**) The model showing the probe inserted 10 mm deep into the MUT, with its input port outside the MUT. All measurements in millimeters.

The basic model of the probe is shown in Figure 2a, as a segment of a coaxial line. At the upper end of the probe, the waveguide port was placed, allowing the measurement of the reflection coefficient. The lower end of the probe was the probe tip, i.e., the open end used for measuring the MUT permittivity. The original slim-form probe is 200 mm long from the open end to the connector. This separates the open end from the connector and the VNA cable (Figure 1), minimizing both the mechanical and the electromagnetic influence of the bulky attachment on the measurement occurring in the MUT. The sufficient probe length also provides the mechanical support for attaching the probe to the stand and keeping it mechanically stabilized (Figure 1). Furthermore, it also allows the insertion of the probe in tall and narrow containers. All these features were irrelevant for the simulations; thus, we minimized the length of the probe model to reduce the simulation burden. We set our model to the length of 11 mm to allow the 10 mm insertion into the MUT, which modeled the actual physical measurement setup within the MUT. The remaining length outside the MUT was arbitrarily chosen as 1 mm to provide a very short segment to which the input port was attached, 1 mm above the MUT border (Figure 2b).

Our simulation model of the slim-form probe exploited the fact that the slim-form probe dimensions conform to the geometry of a standard RG405 semi-rigid coaxial cable. According to, e.g., [39], these are: central conductor diameter 0.51 mm, dielectric diameter 1.68 mm, and shield outer diameter 2.18 mm, with PTFE (Teflon) as the dielectric. In this study, PTFE relative permittivity was set to $\varepsilon_{r}$ = 2.08. PTFE loss tangent was set to zero, as the realistic loss tangent of PTFE is extremely small anyway, and the shortness of the line further reduces any significant losses in the coaxial line. The metallic parts, i.e., the central and the outer conductor, were modeled as perfect electric conductor (PEC). Using PEC as a material with infinite conductivity accelerated the simulations with respect to using realistic metallic materials. We initially compared the simulations of the probe model using PEC to those using metallic materials such as copper to observe if any difference exists in the results. The results were practically identical, concluding that PEC could be used further on, as an efficient approximation of metallic materials as its conductivity is so large that it can be approximated with infinity. After the initial simulations, the central conductor diameter was finely tuned to 0.50465 mm which yielded the accurate characteristic impedance of the coaxial line of 50 Ω ± 0.01 Ω both in FEKO and in CST. This was the basic probe model which also served to simulate the open end without MUT, which was needed for the calibration.

MUT was added into the model as a vertical cylinder, 29 mm high and 37 mm in diameter, centered around the probe, with the probe inserted 10 mm deep into the cylinder (Figure 2b). The complex permittivity of the MUTs was set to their Debye and Cole–Cole values (water and saline solutions, respectively), previously calculated from 500 MHz to 18 GHz, as explained in Section 2.5. Both FEKO and CST allow the user to define the dispersive material by a table of $\varepsilon_{r}^{\prime }$ and $\varepsilon_{r}^{\prime \prime }$ frequency points.

#### 2.7.2. Frequency Domain: Method of Moments in FEKO

The open-ended coaxial probe inserted in MUT was modeled and simulated using method of moments (MoM) implemented in electromagnetic simulation software package FEKO (Altair Engineering, Inc., Troy, MI, USA) [34]. The method of moments is the default and the most accurate method provided by FEKO, implementing a full wave solution of Maxwell’s integral equations in the frequency domain. MoM is an open domain method, i.e., it solves the problems involving radiation sources in free space. Accordingly, there are no problem boundaries to be defined but only the structure itself. Consequently, only the structure is discretized and not the space around it.

The structure volume elements, both metallic and dielectric, were defined by their surfaces, discretized by triangles. Modeling of dielectric media in MoM formulation was performed using the surface equivalence principle (SEP) enabled in FEKO. SEP introduces equivalent electric and magnetic currents on the surface of arbitrarily shaped closed dielectric bodies.

**Figure 3:**
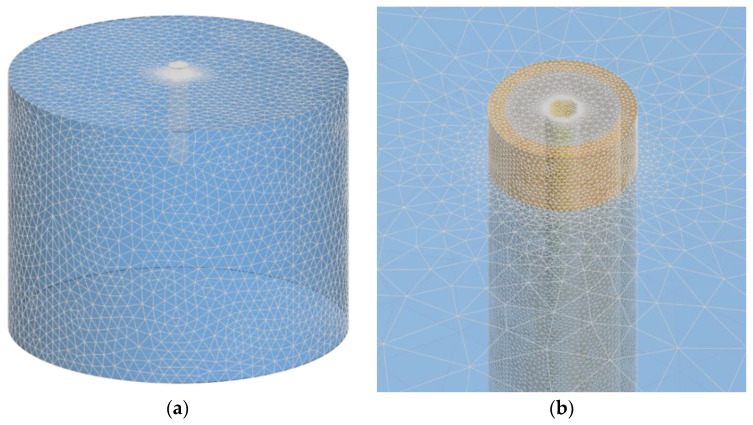
(**a**) FEKO model mesh (probe in MUT). (**b**) Mesh detail around the probe input port.

The frequency range of interest (500 MHz to 18 GHz) was covered by running simulations at discrete frequency points, from 500 MHz to 1 GHz with 100 MHz step, and from 1 to 18 GHz with 1 GHz step. The mesh size increased with the frequency and model complexity. After testing several grades of mesh refinement for the results convergence, we found the optimum mesh resulting with max. ~75k triangles at 18 GHz for the full model of the probe and MUT (Figure 3).

#### 2.7.3. Time Domain: Finite Integration Technique in CST

Aiming to additionally validate the electromagnetic simulation results with a different numerical method, we simulated the same models using a time-domain solver. Time-domain analysis not only complements the frequency-domain study but is also generally suitable and potentially less time-consuming for broadband problems such as this one. The Finite Integration Technique (FIT) implemented in electromagnetic simulation software package CST Studio Suite (Dassault Systèmes, Vélizy-Villacoublay, France) [35] was used as the numerical solver.

**Figure 4:**
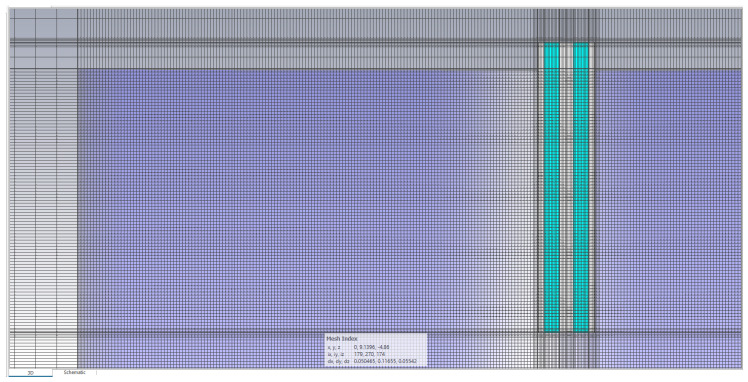
CST model mesh cross-section, truncated on the right and on the bottom to ensure the visibility of the important features of the mesh, showing the cross-section of the whole probe and the upper-left part of MUT (compare to Figure 2b) surrounded by the model box.

Mesh was based on hexahedra. The mesh density was increased near the model, as opposed to the model box edges where the boundary conditions were set as a perfectly matched layer. The probe itself consisted of 2.59 million mesh cells, while the entire model consisting of the probe immersed in MUT had between 31.8 and 33.7 million mesh cells, adequately representing the structure (Figure 4).

For S-parameter calculations, the excitation signal for the transient analysis is defined by a Gaussian shape for which the Fourier spectrum entirely covers the defined frequency band of interest. The accuracy of results was set to −40 dB, and the maximum solver duration was set to 20 pulses. The set accuracy resulted in a total simulation time of just over 10 min for the probe only, 50 min for model with water as a MUT, and between 116 min and 163 min for the saline solutions.

### 2.8. Capacitive-Load Model

The probe open end inserted in MUT terminates the coaxial line, and the probe tip can thus be regarded as terminated by the load admittance *Y*, which inherently depends on the MUT permittivity $\varepsilon_{r}$. In order to derive the MUT relative permittivity $\varepsilon_{r}$ from the reflection coefficient *Γ* measured at the probe input port, the function $\varepsilon_{r}$ = *f*(*Γ*) needs to be determined.

In general, the reflection coefficient at the line end is the function of the line termination load admittance *Y*, given the fixed characteristic admittance of the transmission line *Y*_0_:

(11) $$\Gamma_{\mathrm{MUT}}=\frac{Y_{0}-Y}{Y_{0}+Y}.$$

Equation (11) actually describes the reflection coefficient *Γ*_MUT_ at the probe open end, where the incident wave travelling inside the probe reflects from the interface with the MUT. On the other hand, VNA can only measure the reflection coefficient *Γ* at the probe input port (see Figure 2a). Assuming a lossless transmission line along the probe length, perfectly matched to the VNA, the two reflection coefficients differ only by their phase due to the phase shift from the probe tip to the probe input port, according to the following relation:

(12) $$\Gamma =\Gamma_{\mathrm{MUT}}\frac{1-\mathrm{jtg}\beta l}{1+\mathrm{jtg}\beta l},$$

where the fraction on the right describes the phase shift of the reflection coefficient in a lossless transmission line (i.e., along the probe) having a length of *l*, where *β* = 2 π/*λ*, and *λ* is the wavelength inside the line. The assumption of a lossless line holds when the probe is made of low-loss materials and is too short for any significant losses to occur. However, if the line has its own imperfections in terms of matching and losses, it has to be considered as a two-port network with its scattering matrix having parameters *S*_11_, *S*_12_, *S*_21_, and *S*_22_. At this point, for the sake of simplicity, Equation (12) stands as the special case of a scattering matrix of a matched lossless transmission line having *S*_11_ = *S*_22_ = 0 and *S*_12_ = *S*_21_ = e^−j*βl*^. After establishing the dependencies *Γ* = *f* (*Γ*_MUT_) in (12) and *Γ*_MUT_ = *f* (*Y*) in (11), the last dependency to be established is *Y* = *f* ($\varepsilon_{r}$), in order to finally determine the function $\varepsilon_{r}$ = *f* (*Γ*).

The function *Y* = *f* ($\varepsilon_{r}$) was determined by modeling the admittance of the probe end terminated by MUT. The model complexity can vary from the simplest one, a pure capacitive-load model *Y* = j*ωC*, to more complex ones including other components of the admittance. It has been shown in prior studies [26,47,48,49] that the simple capacitive load successfully models the actual load if the frequencies are not as high as to cause radiation from the probe into the MUT. Accordingly, in this model, the only physical phenomenon taken into account at the probe tip was the purely reactive electric field closing between the two conductors of the coaxial cable, forming the purely capacitive load. This is commonly satisfied within the nominal frequency range of a coaxial probe, which is anyhow limited at the high end by the occurrence of the higher modes of EM wave propagation in the coaxial line. Therefore, we used the simple capacitive-load model consisting of two capacitances:

(13) $$Y=j\omega C_{f}+j\omega \varepsilon C_{0}=j\omega C_{f}+j\omega \varepsilon_{0}\varepsilon_{r}C_{0}$$

where *C***_f_** is the fringing capacitance occurring within the probe dielectric and thus independent of MUT, and ε*C*_0_ is the capacitance arising from the electric field lines closing through the MUT (i.e., describing the electric energy stored in the MUT) and thus dependent on the MUT permittivity ε = $\varepsilon_{0}\varepsilon_{r}$. Since the permittivity $\varepsilon_{r}$ is a complex quantity as shown in (1), the admittance consists of both conductance and susceptance, despite calling this model a “capacitive-load model”:

(14) $$Y=j\omega C_{f}+j\omega \varepsilon_{0}\left(\varepsilon_{r}^{\prime }-j\varepsilon_{r}^{\prime \prime }\right)C_{0}=\omega \varepsilon_{0}\varepsilon_{r}^{\prime \prime }C_{0}+j\omega \left(C_{f}+\varepsilon_{0}\varepsilon_{r}^{\prime }C_{0}\right)=G+jB\;\;\;.$$

The conductance thus describes the losses arising from the imaginary part of the MUT dielectric permittivity (as previously explained in Section 2.2).

It is obvious from (13) and (14) that *Y* = *f* ($\varepsilon_{r}$), and vice versa, $\varepsilon_{r}$ = *f* (*Y* ), and from (11) and (12) that *Γ* = *f* (*Y* ), and vice versa, *Y* = *f* (*Γ*); thus, there must exist a function $\varepsilon_{r}$ = *f* (*Γ*), which can be used to calculate the MUT relative permittivity $\varepsilon_{r}$ from the measured reflection coefficient *Γ*. Solving this function, according to the above given model, requires knowing the three unknowns which depend on the exact construction geometry of the probe: *C*_0_, *C***_f_**, and the probe length *l*. This can be resolved by measuring the reflection coefficient for three known loads, as is commonly done by probe calibration, as mentioned in Section 2.3. This was the reasoning for the perfectly matched lossless probe.

Using the calibration data for three loads, the function $\varepsilon_{r}$ = *f* (*Γ*) can actually be determined and expressed even without the need to determine the said unknowns. Such a procedure is especially useful for a general case of the scattering matrix, applicable to the realistic probes being neither perfectly matched nor lossless. Still, the same model can be used for the load admittance: a simple capacitive load described by (13). The calculation procedure has already been shown in the literature [47,48,49], where the probe was considered as a two-port network with its scattering matrix. The resulting function $\varepsilon_{r}$ = *f* (*Γ*), in accordance with [48,49] is:

(15) $$\varepsilon_{r}=\frac{A_{1}\Gamma -A_{2}}{A_{3}-\Gamma }\;\;\;.$$

All the unknowns, including the scattering parameters in the general case, are contained within three unknowns *A*_1_, *A*_2_, and *A*_3_, which are determined by calibration using three loads. The usual three loads for calibration are open, short, and a known liquid with relative permittivity $\varepsilon_{L}$, producing the reflection coefficients *Γ*_O_, *Γ*_S_, and *Γ*_L_, respectively, during the calibration procedure. The unknowns *A*_1_, *A*_2_, and *A*_3_ are then found from the calibration as (in accordance with [48,49]):

(16) $$A_{1}=\frac{\varepsilon_{L}\left(\Gamma_{S}-\Gamma_{L}\right)-\left(\Gamma_{S}-\Gamma_{O}\right)}{\Gamma_{L}-\Gamma_{O}}\;\;\;,$$

(17) $$A_{2}=\frac{\varepsilon_{L}\Gamma_{O}\left(\Gamma_{S}-\Gamma_{L}\right)-\Gamma_{L}\left(\Gamma_{S}-\Gamma_{O}\right)}{\Gamma_{L}-\Gamma_{O}}\;\;\;,$$

(18) $$A_{3}=\Gamma_{S}\;\;\;.$$

All the quantities in Equations (15)–(18) are complex values.

The calibration was performed in this study throughout the frequency span of the measurements, calculating the unknowns *A*_1_, *A*_2_, and *A*_3_ at each frequency point. This enabled us to calculate the permittivity using Equation (15) at each frequency point. 

Besides performing the calculations (15)–(18) in the measurement part of the study, the same calculations were also used for postprocessing in the simulation part of the study. This postprocessing thus concluded the simulation of the complete measurement process: from modeling the probe and the MUT and obtaining the reflection coefficient for various MUTs by simulations to postprocessing using the simple capacitive-load model.

## 3. Results

### 3.1. Measurement Results

**Figure 5:**
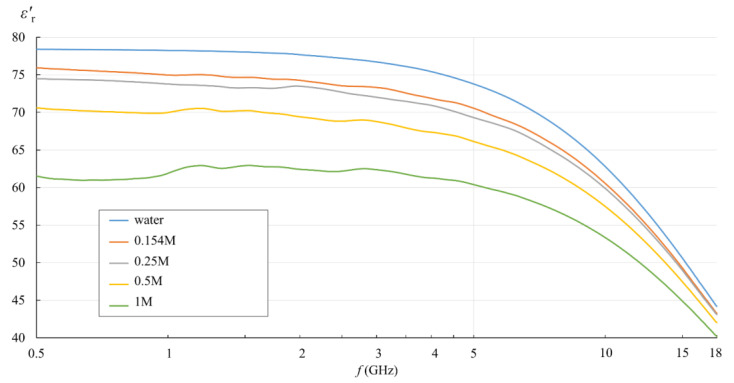
Measured $\varepsilon_{r}^{\prime }$ of water and saline solutions, as obtained from the measurement software (frequency in logarithmic scale).

**Figure 6:**
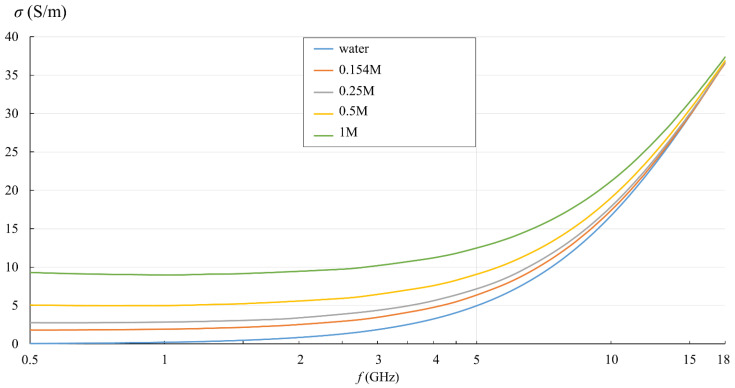
Measured *σ* of water and saline solutions, calculated from $\varepsilon_{r}^{\prime \prime }$ (frequency in logarithmic scale).

**Figure 7:**
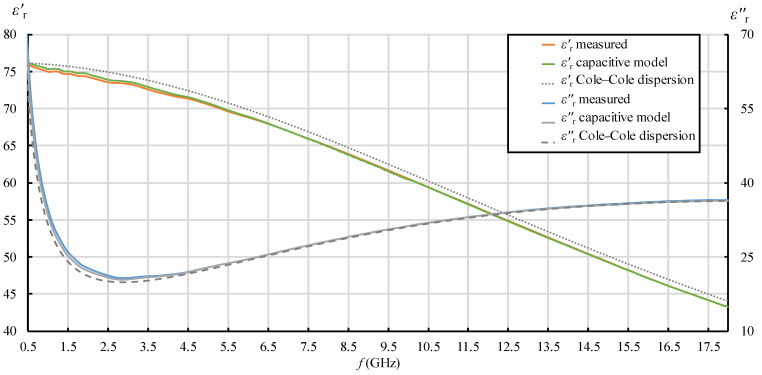
$\varepsilon_{r}^{\prime }$ and $\varepsilon_{r}^{\prime \prime }$ for 0.154M (physiological) saline: measurement result as a final value obtained from the measurement software (“measured”); measurement result obtained by postprocessing the reflection coefficient using the simple capacitive-load model (“capacitive model”) and dispersion model (“Cole–Cole dispersion”).

**Figure 8:**
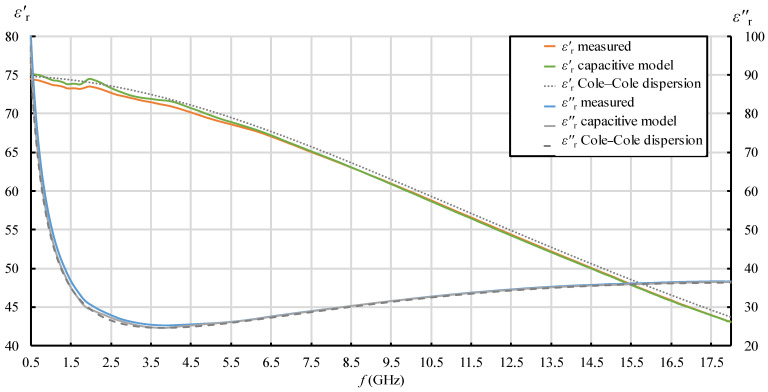
$\varepsilon_{r}^{\prime }$ and $\varepsilon_{r}^{\prime \prime }$ for 0.25M saline: measurement result as a final value obtained from the measurement software (“measured”); measurement result obtained by postprocessing the reflection coefficient using the simple capacitive-load model (“capacitive model”) and dispersion model (“Cole–Cole dispersion”).

**Figure 9:**
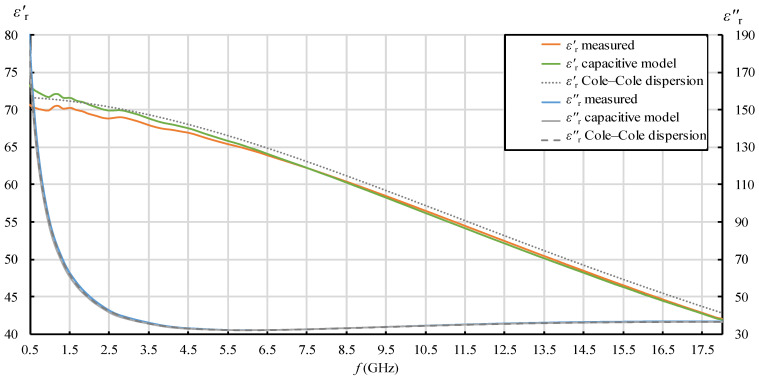
$\varepsilon_{r}^{\prime }$ and $\varepsilon_{r}^{\prime \prime }$ for 0.5M saline: measurement result as a final value obtained from the measurement software (“measured”); measurement result obtained by postprocessing the reflection coefficient using the simple capacitive-load model (“capacitive model”) and dispersion model (“Cole–Cole dispersion”).

**Figure 10:**
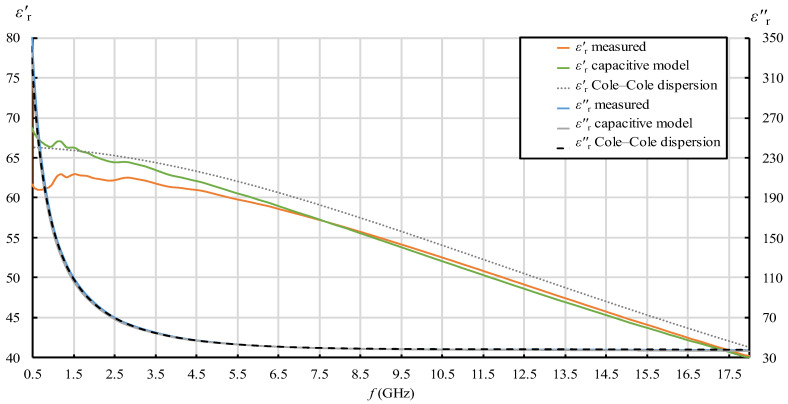
$\varepsilon_{r}^{\prime }$ and $\varepsilon_{r}^{\prime \prime }$ for 1M saline: measurement result as a final value obtained from the measurement software (“measured”); measurement result obtained by postprocessing the reflection coefficient using the simple capacitive-load model (“capacitive model”) and dispersion model (“Cole–Cole dispersion”).

**Figure 11:**
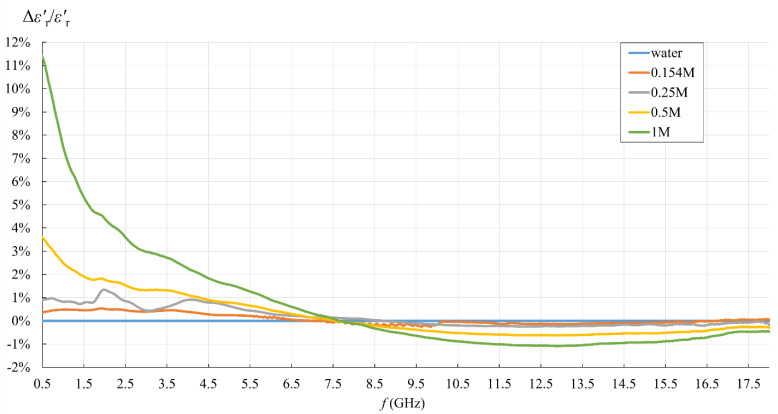
$\varepsilon_{r}^{\prime }$ relative error of the simple capacitive-load model with respect to the measurement result obtained from the measurement software, for water and saline solutions with different NaCl concentrations.

**Figure 12:**
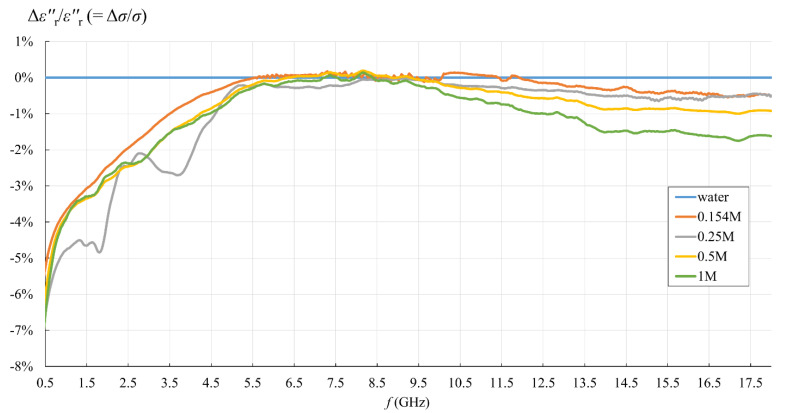
$\varepsilon_{r}^{\prime \prime }$ relative error of the simple capacitive-load model with respect to the measurement result obtained from the measurement software, for water and saline solutions with different NaCl concentrations.

Figure 5, Figure 6, Figure 7, Figure 8, Figure 9, Figure 10, Figure 11 and Figure 12 show the results of the first part of the study, described in Section 2.1.1. The measurement results are drawn, implementing a moving average with the frequency span of ±125 MHz at each frequency point to smooth the lines by eliminating the small fluctuations that do not reflect the actual result fluctuations but are caused by the imperfections in the measurement setup.

Figure 5 and Figure 6 show the measurement results for $\varepsilon_{r}^{\prime }$ and *σ* calculated by (3). They are displayed in the logarithmic scale for easier comparison with the published studies of NaCl solutions in water.

In Figure 5 and Figure 6, the effect of the ionic concentration in the saline solutions can be observed. $\varepsilon_{r}^{\prime }\;$ decreased with the ionic concentration, shifting the whole curve downwards with respect to deionized water. Figure 6 shows the equivalent conductivity *σ* as defined by (3). The conductivity dominated the low frequency range and showed different values for different saline concentrations. At higher frequencies, the equivalent conductivity is a consequence of dielectric losses only, and thus converged towards deionized water.

In Figure 7, Figure 8, Figure 9 and Figure 10, each quantity ($\varepsilon_{r}^{\prime }$ and $\varepsilon_{r}^{\prime \prime }$) is represented with three lines:

- Measurement result as a final value obtained from the measurement software (where the postprocessing of the measured reflection coefficient was embedded in the measurement software);
- Measurement result obtained by postprocessing of the reflection coefficient using the simple capacitive-load model;
- Dispersion model (Debye dispersion for water and Cole–Cole dispersion for saline solutions).

Dispersion models are depicted in Figure 7, Figure 8, Figure 9 and Figure 10 just to show the expected permittivity values for the measured MUTs, but they are not the focus of this part of the study. The dispersions were calculated according to the description given in Section 2.5. It can be observed in Figure 7, Figure 8, Figure 9 and Figure 10 that the measured values slightly and systematically differed from Cole–Cole dispersion models (especially visible in Figure 7, Figure 8, Figure 9 and Figure 10 for $\varepsilon_{r}^{\prime }$), more so as the ionic concentration increased. There might be several reasons for this. First, the dispersion models are not absolutely accurate. The models just approximate the realistic permittivity of the saline. Next, the model parameters depend on the temperature, where slight deviations are possible. Although the MUTs were stabilized in the water bath at 25 °C, the sample temperature slightly fluctuated around this value during its handling out of the bath and during the measurements. Furthermore, and most likely, the measurement accuracy deteriorates as MUT permittivity is further from the reference liquid used in the calibration process (which was deionized water). Anyway, the accuracy of the slim-form probe, described in Section 2.4, was itself declared at ±10%, and the systematic difference between the measured values and the dispersion models was within this declared accuracy. Accordingly, a slight difference between the measured results and the dispersion models was not of interest in this part of the study. Here, we just regarded the results obtained from the measurement software as the accurately measured values (hence as reference values for comparisons), ignoring their deviation from the dispersions. Therefore, the difference between the result obtained by postprocessing using the simple capacitive-load model and the accurate result can be considered as error. The relative error of the simple capacitive-load model with respect to the accurate value can be calculated for $\varepsilon_{r}^{\prime }$ and $\varepsilon_{r}^{\prime \prime }$ as:

(19) $$\varepsilon_{r}^{\prime }\;\mathrm{relative}\;\mathrm{error}\;\left[\%\right]=\frac{\varepsilon_{r\left(\mathrm{capacitive}\;\mathrm{model}\right)}^{\prime }-\varepsilon_{r\left(\mathrm{measured}\right)}^{\prime }}{\varepsilon_{r\left(\mathrm{measured}\right)}^{\prime }}\cdot 100\%\;\;,$$

(20) $$\varepsilon_{r}^{\prime \prime }\;\mathrm{relative}\;\mathrm{error}\left[\%\right]=\frac{\varepsilon_{r\left(\mathrm{capacitive}\;\mathrm{model}\right)}^{\prime \prime }-\varepsilon_{r\left(\mathrm{measured}\right)}^{\prime \prime }}{\varepsilon_{r\left(\mathrm{measured}\right)}^{\prime \prime }}\cdot 100\%=\sigma \;\mathrm{relative}\;\mathrm{error}\left[\%\right]\;\;.$$

Due to equivalent conductivity *σ* being directly proportional to $\varepsilon_{r}^{\prime \prime }$ according to (3), $\varepsilon_{r}^{\prime \prime }$ relative error is equal to *σ* relative error. These relative errors are presented in Figure 11 and Figure 12. Both $\varepsilon_{r}^{\prime }$ and $\varepsilon_{r}^{\prime \prime }$ relative errors equaled zero for water, as the water was used as the calibration standard both for the simple capacitive-load model and for the measurement software.

Figure 7, Figure 8, Figure 9, Figure 10, Figure 11 and Figure 12 show interesting features when focusing on the comparison between the postprocessing methods, which was the research aim of this study. The most pronounced difference occurred in the lower part of the frequency span, below ca. 7.5 GHz, where the simple capacitive-load model systematically yielded slightly larger $\varepsilon_{r}^{\prime }$ than the actually measured value. The difference increased towards the low frequencies, which suggests that the difference between the models is inversely proportional to frequency at low frequencies for $\varepsilon_{r}^{\prime }$. The difference also systematically increased with the ion concentration in the saline, suggesting that the error is connected to ionic polarization at the probe electrode surface. Due to the ionic origin of this phenomenon, it was more prominent at low frequencies and diminished as the frequency increased. However, comparing the measurement results with the dispersions, we concluded that the electrode polarization effect is not so prominent within the specified working frequency range of the slim-form probe. In any case, this low-frequency phenomenon requires additional components in (12) to model the load accurately, which are not included in the simple capacitive-load model. The manufacturer does not declare whether their measurement software models this effect or applies any corrections during postprocessing. Most likely, their measurement results suffer from the polarization error as well. Nevertheless, by regarding their measurement result as the accurate reference in this study, Figure 11 shows that the $\varepsilon_{r}^{\prime }\;$relative error magnitude of the capacitive model stayed within ca. 0.5% for the physiological saline, around 1% for 0.25M saline, less than 4% for 0.5M saline, and exceeded 11% for 1M saline at the lowest frequency.

Figure 11 also shows that the frequency of 7.5 GHz was a cross-over point, where the difference between the models diminished and then changed its sign: above 7.5 GHz, the simple capacitive-load model systematically yielded lower $\varepsilon_{r}^{\prime }$ than the actually measured value. However, the error magnitude stayed within ca. 1% even for the highest NaCl concentration and practically diminished towards the upper limit of the frequency span, overall suggesting a negligible error.

When it comes to $\varepsilon_{r}^{\prime \prime }$, the situation was somewhat different. Although the difference is barely visible in Figure 7, Figure 8, Figure 9 and Figure 10 due to the y-axis scale, it is more clearly visible in Figure 12 where the difference again occurred in the lower part of the frequency span below ca. 5.5 GHz but had the opposite sign: the simple capacitive-load model systematically yielded slightly lower $\varepsilon_{r}^{\prime \prime }$ than the actually measured value. Again, the error increased towards the low frequencies, reaching the maximum magnitude of almost 7% at the lowest frequency, suggesting that the $\varepsilon_{r}^{\prime \prime }\;$relative error is inversely proportional to frequency at low frequencies. However, the $\varepsilon_{r}^{\prime \prime }\;$error at low frequencies apparently did not significantly depend on the ion concentration in the saline. Between ca. 5.5 GHz and 9 GHz, there was no difference between the models. Above 9 GHz, the difference appeared again, and the capacitive-load model started systematically yielding lower $\varepsilon_{r}^{\prime \prime }\;$than the actually measured value. The magnitude of this error was not significant below 18 GHz: <1% for all concentrations lower than 1M and <2% for the highest NaCl concentration of 1M. It is worth noting that the error slightly increased with frequency. This might be connected with the appearance of radiation from the probe end, when the wavelength decreases and becomes comparable to the probe tip dimensions. However, due to the frequency limit of our setup, we were not able to determine the significance of this error above 18 GHz.

Figure 11. $\varepsilon_{r}^{\prime }$ relative error of the simple capacitive-load model with respect to the measurement result obtained from the measurement software, for water and saline solutions with different NaCl concentrations.

### 3.2. Simulation Results with the Simple Capacitive-Load Model

**Figure 13:**
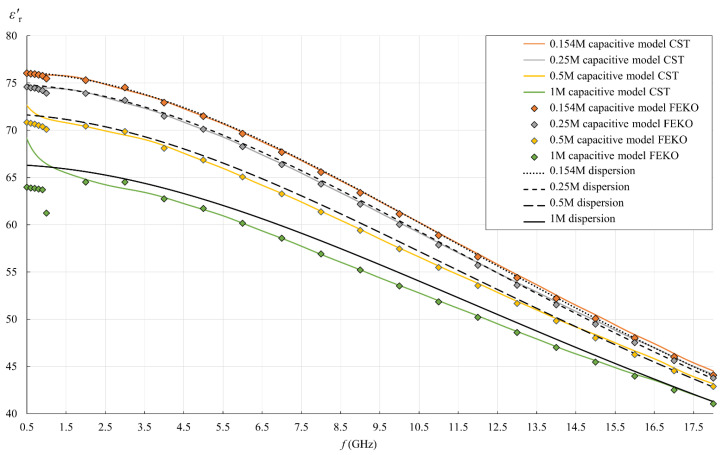
$\varepsilon_{r}^{\prime }$ for all saline solutions: simulation results obtained by postprocessing the reflection coefficient using the simple capacitive-load model (“capacitive model”), where the reflection coefficient was obtained by simulations either in CST (colored lines) or in FEKO (discrete points); Cole–Cole dispersion model (“dispersion”), which was the set $\varepsilon_{r}^{\prime }$ value for MUT in simulations (black lines).

**Figure 14:**
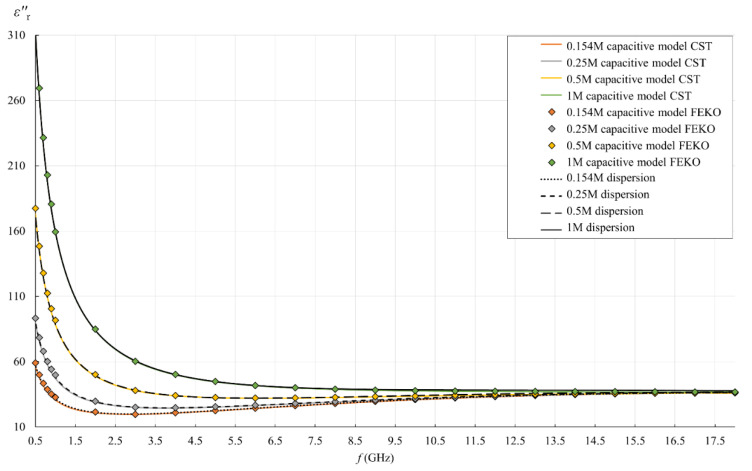
$\varepsilon_{r}^{\prime \prime }$ for all saline solutions: simulation result obtained by postprocessing the reflection coefficient using the simple capacitive-load model (“capacitive model”), where the reflection coefficient was obtained by simulations either in CST (colored lines) or in FEKO (discrete points); Cole–Cole dispersion model (“dispersion”), which was the set $\varepsilon_{r}^{\prime \prime }$ value for MUT in simulations (black lines).

**Figure 15:**
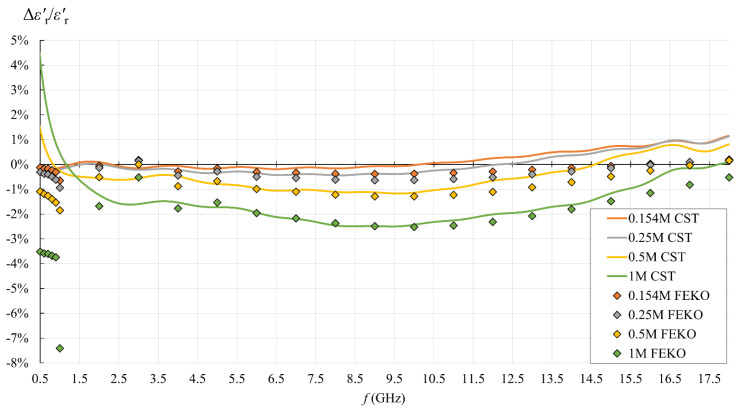
$\varepsilon_{r}^{\prime }$ relative error of the simple capacitive-load model (FEKO: discrete points; CST: solid lines) with respect to the set $\varepsilon_{r}^{\prime }$ value.

**Figure 16:**
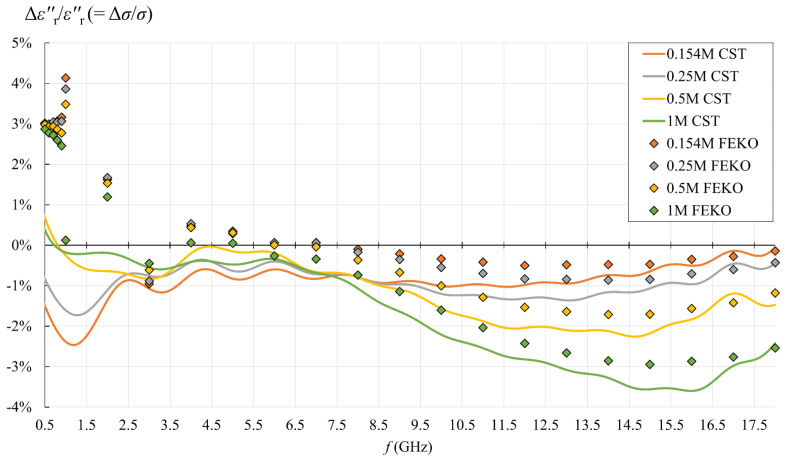
$\varepsilon_{r}^{\prime \prime }$ relative error of the simple capacitive-load model (FEKO: discrete points; CST: solid lines) with respect to the set $\varepsilon_{r}^{\prime \prime }$ value.

Figure 13, Figure 14, Figure 15 and Figure 16 show the results of the second part of the study, described in Section 2.1.2.

Figure 13 and Figure 14 show the simulation results for $\varepsilon_{r}^{\prime }$ and $\varepsilon_{r}^{\prime \prime }$, as obtained by postprocessing of the reflection coefficient using the simple capacitive-load model. The reflection coefficient was obtained by simulations using FEKO and CST. FEKO is a frequency-domain method; therefore, the results are represented in Figure 13, Figure 14, Figure 15 and Figure 16 by discrete frequency points for which the simulation was performed (as described in Section 2.7.2). CST is a time-domain method, and its results continuously covered the frequency span of the simulations (colored lines in figures). Both FEKO and CST results can be compared to the dispersion models, also shown in figures (dotted, dashed, and solid black lines).

In this simulation part of the study, the dispersion model represents the accurate permittivity, as this was the MUT permittivity value actually set in each simulation model. By comparing the simulation results to the dispersions, we can observe the validity of the overall simulation process, consisting of both the electromagnetic simulations and the postprocessing using the capacitive model. The postprocessing of the reflection coefficient should revert the simulation result right back to the permittivity initially set in the model.

Figure 13 and Figure 14 clearly show the similarities and differences between the used simulation packages (CST and FEKO) and the associated methods, which was one of the research aspects of this study. The most obvious observation refers to their performance and output: the time-domain method (in CST) resolved the frequency range in just one simulation and provided a continuous curve. On the other hand, the frequency-domain method (in FEKO) required a large number of simulations to cover the same range, which is time-consuming and produces only the discrete points, thus potentially omitting the phenomena occurring between these frequency points. This makes the frequency-domain methods inferior when it comes to studying the broadband response, like in this study. Moreover, when the frequency span is so wide as in this study, it cannot be covered by a single model mesh in FEKO, i.e., the mesh parameters must be adapted to different frequencies, which is again time-consuming and causes additional errors if the model mesh is not adjusted properly. This might be the reason why FEKO results deviated from a smooth line at low frequencies (observable in Figure 13): it was obvious that discrete points do not form a smooth line below 2 GHz. The model mesh was indeed changed across that range. We did ensure the validity of the simulation results by refining the mesh until the result converged. Nevertheless, the final plot of the results shows discontinuities under 2 GHz. In the same range, $\varepsilon_{r}^{\prime }$ by FEKO fell lower than both the dispersion and CST. Perhaps FEKO models could be further refined to provide better agreement. However, this disagreement was quite small, while CST provided slightly better agreement with dispersions at low frequencies and produced a continuous wideband response. Accordingly, there was no rationale for further refinement of FEKO models.

Figure 15 and Figure 16 show the relative error between the CST simulation, FEKO simulation, and dispersion, defined as:

(21) $$\varepsilon_{r}^{\prime }\;\mathrm{relative}\;\mathrm{error}\left[\%\right]=\frac{\varepsilon_{r\left(\mathrm{capacitive}\;\mathrm{model}\right)}^{\prime }-\varepsilon_{r\left(\mathrm{dispersion}\right)}^{\prime }}{\varepsilon_{r\left(\mathrm{dispersion}\right)}^{\prime }}\cdot 100\%\;\;,$$

(22) $$\varepsilon_{r}^{\prime \prime }\;\mathrm{relative}\;\mathrm{error}\left[\%\right]=\frac{\varepsilon_{r\left(\mathrm{capacitive}\;\mathrm{model}\right)}^{\prime \prime }-\varepsilon_{r\left(\mathrm{dispersion}\right)}^{\prime \prime }}{\varepsilon_{r\left(\mathrm{dispersion}\right)}^{\prime \prime }}\cdot 100\%=\sigma \;\mathrm{relative}\;\mathrm{error}\left[\%\right]\;.$$

The agreement between FEKO, CST, and dispersion depends on both frequency and ionic concentration. As seen in Figure 15, at lower concentrations of 0.154M and 0.25M, FEKO and CST agreed very well on $\varepsilon_{r}^{\prime }$, and also with the dispersion within the relative error of <1%. At 0.5M, FEKO and CST followed each other tightly above 2 GHz, while for both, the error magnitude only slightly exceeded 1% throughout most of the frequency range and reached the maximum of ca. 2% for FEKO at 1 GHz. For 1M saline, FEKO and CST followed each other tightly above 4 GHz where the maximum error magnitude reached ca. 2.5%. At lower frequencies, CST error increased to >4% maximum, while FEKO error magnitude reached the maximum of >7% at 1 GHz.

For $\varepsilon_{r}^{\prime \prime }$, two frequency ranges can be observed, below and above 3 GHz. Below 3 GHz, FEKO and CST diverged, as FEKO exhibited positive error going up to ca. 3–4% maximum, and CST error was negative with magnitude up to ca. 2–3% maximum. The error below 3 GHz apparently did not strongly depend on the concentration. Above 3 GHz, FEKO and CST converged with each other above within 1% error for all concentrations. However, with respect to the dispersion, the error above 3 GHz for both of them increased with NaCl concentration. At lower concentrations of 0.154M and 0.25M, the error magnitude above 3 GHz stayed within 1% and ca. 1.5%, respectively. At 0.5M, the error magnitude above 3 GHz reached the maximum of slightly over 2%, while for 1M, the maximum was ca. 3.5%.

## 4. Discussion

### 4.1. Measurement Results with Respect to the Expected Saline Permittivity

Saline solutions are convenient MUTs to study the effect of ionic concentration on dielectric permittivity. The lowest concentration studied here (0.154M) is the so-called physiological saline [45], thus very relevant for translating the results of this study to biological materials with high water content. Furthermore, permittivity of NaCl solutions in water has been measured and modeled in the literature, e.g., [44,50,51,52,53]. However, this study does not perfectly overlap with those studies concerning the exact NaCl concentrations, saline temperature, and frequency range. The most similar parameters are in studies [44,53], bringing the results for the concentrations 0.2M, 0.5M, and 1M up to 20 GHz at 20 °C, and for 0.154M up to 50 GHz at 23 °C, respectively. Lots of similarities and similar trends can be observed. At similar concentrations and temperatures, $\varepsilon_{r}^{\prime }$, $\varepsilon_{r}^{\prime \prime }$, and *σ* are very similar in magnitude to the values in the literature and exhibit the exact same trends. The whole $\varepsilon_{r}^{\prime }\;$lines in our study were systematically shifted slightly downwards and the whole *σ* lines slightly upwards with respect to other studies, which might be attributed to the slightly higher temperature in our study. However, our results showed the same trend of systematic deviation also from the models in Table 1 (calculated by (7)–(11)), more so as the ionic concentration increased. We are not aware of the cause of this phenomenon, other than those already mentioned in Section 3.1. However, it is worth noting that the model in (7)–(11) is based on the measurement results in [44], whose results are again slightly different from other studies and might also be slightly different from ours for unknown reasons. Nevertheless, considering that the results slightly vary between the previously published studies and that our results’ deviation from the model was very small (less than 2% average error at concentrations lower than 1M), we were not concerned with this, especially since this is not a study on the accurate dielectric properties of saline solutions. Accordingly, we could conclude that our measurement method was validated by fairly accurate results for saline solutions, which was the aim of this comparison.

The next phenomenon to be observed is about the smoothness of $\varepsilon_{r}^{\prime }$, $\varepsilon_{r}^{\prime \prime }$, and *σ* lines. Unlike $\varepsilon_{r}^{\prime \prime }$ and *σ* lines, $\varepsilon_{r}^{\prime }\;$lines were not smooth in the lower frequency range, and the line fluctuation was more observable at higher NaCl concentrations. This was also noticed and commented in [44,53]. In [44], this was commented as instability of the measurement in the presence of high conductivities at frequencies below a few hundred megahertz, and in [53], this was commented to be a consequence of the increased measurement uncertainty due to electrode polarization effect, which is again correlated with conductivity. The formation of electrical double layer at the electrodes due to ionic polarization is a well-known electrochemical effect [54], also described in the context of dielectric measurements, e.g., [15]. The double layer of electrical charges introduces additional capacitive and resistive components into the load seen by the probe. These depend on many factors: MUT composition, electrode material, shape and roughness, frequency, etc. Not only are they difficult to model but they tend to dominate the load impedance at lower frequencies, reducing the probe sensitivity to MUT and creating instabilities. As such, this is the major limiting factor for dielectric probes’ lower frequency limit.

The same phenomenon causes the systematic deviation of $\varepsilon_{r}^{\prime }$ measurement results at low frequencies: measured $\varepsilon_{r}^{\prime }$ exhibits underestimation with respect to the expected trend of the line (the beginning of the line drops towards lower frequencies). This could be observed in our study, especially for higher concentrations of 0.5M and 1M (Figure 9 and Figure 10, respectively), and also in [53] for a low concentration of 0.154M.

### 4.2. Measurement Results with Respect to the Use of Simple Capacitive-Load Model

This topic was the basic motivation for the measurement part of the study. The findings can be summarized in Figure 11 and Figure 12. For the sake of this study, we took the final value obtained from the measurement software as the accurate result, i.e., the reference value for error calculation by Equations (19) and (20). Although this assumption of accuracy might be wrong, or at least has its limitations, there is no apparent reason to doubt that the manufacturer optimized the postprocessing method to their probe. A different approach to this issue would certainly require a separate study in that respect. In any case, if the accuracy of our reference results was jeopardized by the error embedded in the measured reflection coefficient (e.g., due to electrode polarization at low frequencies), the same error would have also been embedded in the result calculated by the capacitive model, as it used the same measured reflection coefficient as the input value. Thus, the errors in the pure measurement process are equal between the models and cancel out when calculating the relative error between the capacitive model and the result obtained from the measurement software.

The capacitive model was rather accurate in the upper frequency range, within ca. 1% relative error for $\varepsilon_{r}^{\prime }$ starting from ca. 6 GHz and within ca. 1.5% relative error for $\varepsilon_{r}^{\prime \prime }$ starting from ca. 4.5 GHz. Therefore, the validity of the capacitive model depends on the acceptable limit for the maximum relative error magnitude in the lower frequency range. Setting this limit to 10%, which corresponds to the declared probe accuracy, made the capacitive model acceptable for all analyzed concentrations up to 1M (the error exceeded 10% only at the lowest frequency for $\varepsilon_{r}^{\prime }$ for 1M). If better accuracy is required, the limit is imposed by $\varepsilon_{r}^{\prime \prime }$ error below 4.5 GHz, reaching almost 7%, and apparently does not depend on the concentration.

From the point of view of biological materials, the interest is focused on the 0.154M saline. Using the simple capacitive-load model for 0.154M saline produced only negligible error, less than 0.5% for $\varepsilon_{r}^{\prime }$ in the entire frequency range and for $\varepsilon_{r}^{\prime \prime }$ in the upper frequency range. The error increased only for $\varepsilon_{r}^{\prime \prime }$ in the lower frequency range, below 4.5 GHz, reaching the maximum magnitude of 5.3%.

Table 2 shows the maximum relative error magnitude, average relative error magnitude, and the standard deviation of the relative error magnitude for all concentrations.

Similar analyses of the capacitive model applicability have been published in [27,28]. In [27], 0.5M and 1M saline solutions were measured with an open-ended coaxial probe of similar dimensions (however, fabricated from a semirigid cable), then the result (i.e., the reflection coefficient) was postprocessed with the capacitive model and other load models (antenna model, virtual line model, and rational function). The capacitive model was found to give a much larger error than in our study in a similar frequency range (50 MHz–20.05 GHz). However, study [27] must be taken with some reservation, as the error was calculated against the theoretical permittivity for saline solutions, which was calculated according to Stogryn formulas for saline permittivity [50]. Stogryn model, although heavily cited in the literature, was partly disproved by Peyman et al. in [44], i.e., the model was observed to create significant and systematic error for larger NaCl concentrations. Actually, the Stogryn model is based on extrapolation from a reduced set of results and thus inherently has its limitations, which were spotted by Peyman et al. If the study [27] used the correct theoretical permittivity, the capacitive model error would be smaller. Furthermore, unlike the usual calibration of the probe at its open end and de-embedding the result using a general case of scattering matrix for the probe to include all imperfections (as in our study, see Section 2.8), in [27], the reflection coefficient measurement plane was at the probe connector, and then the reflection coefficient at the open end was calculated assuming a lossless line and using a theoretical expression for capacitances *C***_f_** and *C*_0_. This procedure could have caused significant errors, which would then be included in their overall result of the capacitive model. Therefore, their conclusions about the error of the capacitive model should not be taken as accurate. The study [28] measured 0.15M saline from 50 MHz–2.6 GHz with a probe wider than ours but used the same procedure as our study: calibration with three loads at the open end, implicitly solving the scattering matrix of a two-port network representing the transmission line between the VNA and the open end. It showed a much smaller error of the simple capacitive-load model than [27], with the error magnitude comparable to ours at the lower frequencies (the study [28] did not reach the turnover frequency), except that our error for $\varepsilon_{r}^{\prime \prime }$ at the lower frequencies was even larger. The trend is also the same: slight overestimation of $\varepsilon_{r}^{\prime }\;$ and underestimation of $\varepsilon_{r}^{\prime \prime }$ at lower frequencies. Therefore, our results are consistent with the study [28].

### 4.3. Simulation Results with Respect to the Use of Simple Capacitive-Load Model

In measurements, we compared the capacitive model to the measurement result, which we regarded as accurate reference permittivity, although this assumption might have been wrong. Here, on the other hand, we had the reference permittivity that we set ourselves in the computer model, so it actually is the accurate permittivity. Another difference from measurements is that the reflection coefficient was obtained here by simulation; thus, its accuracy depends on the simulation accuracy. Therefore, all inaccuracies of the simulation, either due to model validity issues or due to numerical errors, are embedded in the reflection coefficient result, yielding an error that propagates in the permittivity calculated by the capacitive model. Accordingly, the error of the capacitive model vs. the reference permittivity includes not only the imperfections of the capacitive model but also the simulation errors. This might be the cause of discrepancy of trends between CST and FEKO at low frequencies: CST producing overestimation of $\varepsilon_{r}^{\prime }$ and underestimation of $\varepsilon_{r}^{\prime \prime }$, and vice versa for FEKO. In any case, here, the phenomena at low frequencies were not due to electrode polarization, as the simulations are not aware of that effect and do not take it into account.

Table 3 and Table 4 show the maximum magnitude of the relative error, average relative error magnitude, and the standard deviation of the relative error magnitude for all concentrations, for both solvers. The error magnitudes were very similar, although the comparison should be taken with reservation because FEKO errors refer to the discrete frequency points only; thus, the comparison to CST is not straightforward.

Since CST simulation (with FIT) was superior for this analysis over FEKO simulation (with MoM) and provided continuous results over the entire frequency range, only CST results are discussed further.

Figure 15 and Figure 16 show that the capacitive model was rather accurate throughout the frequency range, with the error increasing for higher concentrations and at the lowest frequencies. Setting the acceptable limit for the relative error magnitude to 10%, which corresponds to the declared probe accuracy, made the capacitive model valid for all analyzed concentrations in the entire frequency range. From the point of view of biological materials, the interest is focused on the 0.154M saline. Using the simple capacitive-load model for 0.154M produced only negligible error, less than 1% for $\varepsilon_{r}^{\prime }$ in the entire frequency range and for $\varepsilon_{r}^{\prime \prime }$ in the upper frequency range. The error increased only for $\varepsilon_{r}^{\prime \prime }$ in the lower frequency range, below 3.5 GHz, but stayed within 2.5% magnitude.

A similar simulation study was published in [30]. The same probe was simulated using CST, up to 5 GHz, with two biological MUTs, liver and fat. The liver and fat permittivities were taken from the literature and set in the simulation model. Liver is a more lossy material with higher permittivity than fat, having $\varepsilon_{r}^{\prime }$ of ca. 60 and *σ* of ca. 0.7 S/m at 500 MHz. These values are not very far from the values of the physiological saline and thus are comparable to our study. The magnitude of the average relative error in [30] was quantified at 1.9% for $\varepsilon_{r}^{\prime }$ and 2.1% for $\varepsilon_{r}^{\prime \prime }$ for liver, within the frequency limits of the simulation in [30]. Although the error is higher than in our study, we can regard the studies to be rather consistent.

It is worth noting that the simulation results systematically produced $\varepsilon_{r}^{\prime }$ lower than the dispersion (Figure 13), shifting the whole $\varepsilon_{r}^{\prime }$ curve downwards, more so as the ionic concentration increased. Although the dispersion was not a reference for the measurement study, it is interesting to observe the same phenomenon in measurement results (Figure 7, Figure 8, Figure 9 and Figure 10), both with the capacitive model and with the measured value obtained from the measurement software. This cannot be attributed to the simple capacitive-load postprocessing, as the phenomenon also occurred with the measured value obtained from the measurement software. It also cannot be attributed to any polarization effect, since the simulation does not take polarization into account. We hypothesize that this might be related to the large difference in $\varepsilon_{r}^{\prime \prime }$ between the measured conductive MUTs and the calibration liquid (deionized water), but testing this hypothesis exceeds the scope of this study and will be addressed in future work.

## 5. Conclusions

Our study, consisting of the measurement and the simulation part, showed that the dielectric permittivity measurement method using open-ended coaxial probe in the microwave frequency range can be completely and reliably modeled and simulated using computer electromagnetic modeling and simulations followed by the postprocessing calculations. Furthermore, the study showed that the postprocessing calculations can be performed using the simple capacitive-load model, obtaining very accurate results.

When the simple capacitive-load model was used for postprocessing the measured reflection coefficients, the results were within the typical measurement accuracy of the probe itself. The main deficiency of the model showed up at the lowest part of the probe frequency range, where ionic polarization occurs at the probe electrodes, which is not taken into account by the model. However, the lowest part of the probe frequency range was critical even when using the probe with the manufacturer’s measurement software.

When used for postprocessing the electromagnetic simulation results, the simple capacitive-load model yielded only negligible errors, both for the frequency-domain and for the time-domain solvers. We also concluded that the time-domain solver (FIT in CST) was superior for this analysis over the frequency-domain solver (MoM in FEKO) due to providing continuous results over the entire frequency range with excellent accuracy.

Both in measurements and in simulations, the error of the simple capacitive-load model was proportional to the ionic concentration of the MUT. For the physiological saline, the deviation of the capacitive model from the accurate results was negligible. This is an important conclusion, as our primary research interest is in dielectric measurements of biological materials, and the physiological saline approximates the biological materials with high water content.

All the conclusions are applicable in the studied frequency range, covering the lowest and the middle part of the declared probe frequency range. The highest part of the probe frequency range will be analyzed in future studies.

By validating the simulation of the complete measurement process, consisting of computer electromagnetic modeling and simulations followed by the postprocessing calculations using the simple capacitive-load model, in our future work, we will be able to confidently use this simulation method when studying various dielectric measurement scenarios and the associated phenomena. This will be especially useful for studies on biological tissues, where the realizations of the measurement scenarios can be very demanding, that will thus extremely benefit from an accurate simulation method.

## Figures and Tables

**Table 1:** Cole–Cole parameters of the analyzed saline solutions.

Saline Concentration	0.154M	0.25M	0.5M	1M
$\varepsilon_{s}$	76.22	74.912	71.741	66.398
*τ* [s]	8.033 × 10^−12^	7.930 × 10^−12^	7.686 × 10^−12^	7.297 × 10^−12^
*α*	1.020 × 10^−2^	1.535 × 10^−2^	2.432 × 10^−2^	2.313 × 10^−2^
$\sigma_{i}$ [S/m]	1.543	2.469	4.741	8.691

**Table 2:** Measurement study—relative errors.

	Saline Concentration	0.154M	0.25M	0.5M	1M
$\varepsilon_{r}^{\prime }$	Maximum relative error magnitude	0.5%	1.3%	3.6%	11.3%
	Average relative error magnitude	0.2%	0.3%	0.7%	1.6%
	Standard deviation of the relative error magnitude	0.2%	0.4%	0.9%	2.4%
$\varepsilon_{r}^{\prime \prime }$	Maximum relative error magnitude	5.3%	6.6%	6.1%	6.6%
	Average relative error magnitude	0.6%	1.0%	1.0%	1.2%
	Standard deviation of the relative error magnitude	1.0%	1.4%	1.1%	1.1%

**Table 3:** CST simulation study—relative errors.

	Saline Concentration	0.154M	0.25M	0.5M	1M
$\varepsilon_{r}^{\prime }$	Maximum relative error magnitude	1.1%	1.1%	1.4%	4.2%
	Average relative error magnitude	0.3%	0.4%	0.7%	1.6%
	Standard deviation of the relative error magnitude	0.3%	0.3%	0.3%	0.7%
$\varepsilon_{r}^{\prime \prime }$	Maximum relative error magnitude	2.5%	1.7%	2.3%	3.6%
	Average relative error magnitude	0.9%	0.9%	1.2%	1.8%
	Standard deviation of the relative error magnitude	0.4%	0.3%	0.7%	1.3%

**Table 4:** FEKO simulation study—relative errors.

	Saline Concentration	0.154M	0.25M	0.5M	1M
$\varepsilon_{r}^{\prime }$	Maximum relative error magnitude	0.7%	0.9%	1.8%	7.4%
	Average relative error magnitude	0.2%	0.4%	0.9%	2.4%
	Standard deviation of the relative error magnitude	0.1%	0.2%	0.5%	1.4%
$\varepsilon_{r}^{\prime \prime }$	Maximum relative error magnitude	4.1%	3.9%	3.5%	2.9%
	Average relative error magnitude	1.2%	1.3%	1.5%	1.8%
	Standard deviation of the relative error magnitude	1.3%	1.2%	1.0%	1.1%

## Data Availability

Not applicable.
